# Effect of Tumor Necrosis Factor-*α* on Neutralization of Ventricular Fibrillation in Rats with Acute Myocardial Infarction

**DOI:** 10.1155/2011/565238

**Published:** 2011-04-27

**Authors:** Yu Chen, Qing Zhang, Yu-Hua Liao, Zhe Cao, Yi-Mei Du, Jia-Ding Xia, Hua Yang, Zhi-Jian Chen

**Affiliations:** Department of Cardiology, Wuhan Union Hospital and Institute of Cardiovascular Disease, Tongji Medical College, Huazhong University of Science and Technology, Jiefang Road 1277, Jianghan District, Wuhan 430022, China

## Abstract

The purpose of this study was to explore the effects of tumor necrosis factor-*α* (TNF-*α*) on ventricular fibrillation (VF) in rats with acute myocardial infarction (AMI). Rats were randomly classified into AMI group, sham operation group and recombinant human tumor necrosis factor receptor:Fc fusion protein (rhTNFR:Fc) group. Spontaneous and induced VFs were recorded. Monophasic action potentials (MAPs) among different zones of myocardium were recorded at eight time points before and after ligation and MAP duration dispersions (MAPDds) were calculated. Then expression of TNF-*α* among different myocardial zones was detected. After ligation of the left anterior descending coronary artery, total TNF-*α* expression in AMI group began to markedly increase at 10 min, reached a climax at 20–30min, and then gradually decreased. The time-windows of VFs and MAPDds in the border zone performed in a similar way. At the same time-point, the expression of TNF-*α* in the ischemia zone was greater than that in the border zone, and little in the non-ischemia zone. Although the time windows of TNF-*α* expression, the MAPDds in the border zone and the occurrence of VFs in the rhTNFR:Fc group were similar to those in the AMI group, they all decreased in the rhTNFR:Fc group. Our findings demonstrate that TNF-*α* could enlarge the MAPDds in the border zone, and promote the onset of VFs.

## 1. Introduction

Tumor necrosis factor-*α* (TNF-*α*) is an important inflammatory factor with a wide range of biological effects, such as inflammation, apoptosis, and so forth [1–3]. Recent studies found that it is involved in the cardiomyocyte apoptosis, mediates ventricular remodeling, and affects the normal structure and function of the heart seriously [4, 5]. Acute myocardial infarction (AMI) is one of the most severe cardiovascular diseases. In the USA, over 1 million people experience myocardial infarction every year [6]. During AMI, marked inflammatory response exists in acute ischemic myocardium [7–9]. A variety of inflammatory factors participate in the response, including tumor necrosis factor-*α* (TNF-*α*). It has been demonstrated that ischemic myocardium could greatly express TNF-*α* [10, 11] during AMI. Li showed that TNF-*α* release early in the course of AMI contributes to myocardial injury [12]. Sugano et al. [13, 14] found that soluble TNF-alpha receptor 1 (an antagonist to TNF-*α*) improves cardiac function and reduces infarct size. The effect of TNF-*α* on the arrhythmias remains unclear. Shimoda found that TNF-*α* lever was higher in AMI patients with malignant ventricular arrhythmias than control [15]. Our preclinical studies found that plasma concentrations of TNF-*α* rose in AMI patients and was related to the occurrence of ventricular arrhythmias [16]. Then, our animal experiments confirmed that the increased myocardial expression of TNF-*α* in AMI rats was closely related to the occurrence of ventricular arrhythmias [17, 18]. Ventricular fibrillation is a kind of serious complication of AMI, which is the most common cause of sudden cardiac death [19]. Whether TNF-*α* plays an important role in the occurrence of VFs remains unknown. 

When AMI occurs, in accordance with different degrees of ischemia, myocardium could be divided into the ischemic zone, the nonischemic zone, and the border zone [20]. This study detected expression of TNF-*α* in different regions of ischemic myocardium by immunohistochemistry and real-time fluorescence quantitative PCR, recorded the monophasic action potentials (MAPs) and occurrence of VFs, and then explored the effect of TNF-*α* expression on the occurrence of VFs and the potential mechanisms.

## 2. Materials and Methods

### 2.1. Animal Care

All experimental procedures were approved by the Institutional Authority for Laboratory Animal Care and conformed to the Guide for the Care and Use of Laboratory Animals published by the National Institutes of Health (NIH publication No. 85-23, revised 1985). 

### 2.2. AMI Rat Model

Acute myocardial infarction (AMI) was induced in male Wistar rats weighing 250~300 g. All rats were anesthetized with pentobarbital sodium (30~35 mL/kg) by intraperitoneal injection. Under controlled ventilation, a thoracotomy through a left parasternal 3, 4 intercostal spaces was performed, the pericardium was incised, and the anterior wall of the left ventricle was exposed. Left anterior descending coronary artery (LAD) proximal end was ligated with 6-0 line at the junction of the pulmonary conus and the left atrial appendage, which could induce extensive infarction of left ventricular anterior wall. When the ventricular anterior wall turned to be pale or cyanosed and ECG showed ST-segment elevated, myocardial infarction model succeeded. 

### 2.3. Experimental Groups

Two hundred and forty Wistar rats were randomized into AMI group (*n* = 80), sham-operation group (*n* = 80), and recombinant human tumor necrosis factor receptor:Fc fusion protein (rhTNFR:Fc) group (*n* = 80). Anterior wall myocardial infarction was produced in AMI group by ligating the left anterior descending coronary artery (LAD); there was no ligation but operation in sham-operation group. rhTNFR:Fc group was treated with rhTNFR:Fc (10 mg/kg), a TNF-*α* antagonist, 24 hours before LAD ligation. ECG and spontaneous VFs were observed during the whole experiment. Monophasic action potentials (MAPs) among the ischemic zone, the border zone, and the non-ischemia zone were observed at baseline, 10 min, 20 min, 30 min, 60 min, 3 h, 6 h, and 12 h after ligation, and monophasic action potential duration disperses (MAPDds) were calculated, while VFs were induced by S1S2 programmed electrical stimulation and recorded. Subsequently, expression of TNF-*α* among different zones was detected by histochemistry and real-time fluorescent quantitative PCR.

### 2.4. Recording of MAP and Calculation of Monophasic Action Potential Repolarization Dispersion (MAPDd)

Electrodes were placed on the ischemic zone, the nonischemic zone, and the border zone of the epicardium, and each zone recorded five points. Signal input terminal was connected to the BL-420F biological signal acquisition and processing system (Taimeng, China). The signals were inputted into the computer by the A/D converter. Pacing with 6 Hz at high right atrium, MAPs were recorded at baseline, 10 min, 20 min, 30 min, 60 min, 3 h, 6 h, and 12 h after ligation. After measuring MAPD90 of five points of each zone,

(1) $$\text{MAPDd}={\left(\sqrt{\text{MAPD}90_{1}}-\bar{x}\right)}^{2}+\cdots +\frac{{\left(\text{MAPD}90_{5}-\bar{x}\right)}^{2}}{5}.$$

### 2.5. Recording ECG and Observing the Occurrence of VFs

Spontaneous and induced VFs were recorded with BL-420F biological signal acquisition and processing system during the whole experiment. VFs were induced by the S1S2 programmed electrical stimulation with the Y-2 electric stimulator (Chengdu Instrument Factory, China) at different time points before and after LAD ligation (S1 pulse number: 8; S2 pulse number: 1; initial S1S2 interval: 100 ms; S1S2 interval decreasing: 1 ms; cycle interval: 1 s; cycles: 100).

### 2.6. Immunohistochemistry for TNF-*α*

Fresh myocardial tissues of rats were fixed with formalin, dehydrated, transparent, embedded in paraffin, and then cut into 5–8 *μ*m thick slices. After peroxidase was inactivated by hydrogen peroxide at room temperature for 10 min, the slices were repaired by microwave for 10 min, incubated with BSA at 37°C for 40 min, with a 1 : 200 dilution of goat antirat TNF-*α*/TNFSF1A antibody (R&D Systems, USA) TNF-*α* antibody at 4°C overnight, with a 1 : 100 dilution of biotinylated rabbit antigoat secondary antibody (Boshide, China) at 37°C for 40 min, with avidin-biotin-horseradish peroxidase complexes at 37°C for 60 min, then colored with diaminobenzidine, stained with haematoxylin, and, finally, observed. Selecting five sights at each slice, TNF-*α* expression in every secition was measured and analyzed by HMIAS Series Color Medical Image Analyze System (Champion Image Ltd., China).

### 2.7. Real-Time Quantitative PCR for TNF-*α* mRNA

Myocardial cell total RNA was extracted with TRIzol reagent (Invitrogen, USA). Then, reverse transcription with ReverTra Ace-*α*-kit (TOYOBO Japan), amplifying with SYBR Green mix (TOYOBO Japan), and detecting fluorescence by SLAN Real-time Quantitative PCR Detection System (Hongshi, China). The following oligonucleotides were used as rat sense and antisense primers; upper primer: 5′-CAGCCGATTTGCCATTTCAT-3′, lower primer: 5′-ACGCCAGTCGCTTCACAGAG-3′ (synthesized by Gibco, Grand Island, NY). Thermal cycling conditions were 95°C, 15 s, 56°C, 15 s, 72°C, 45 s, 40 cycles. With housekeeping gene *β*-actin (249 bp) as internal control, the relative quantity of TNF-*α* mRNA was analyzed.

### 2.8. Statistical Analysis

All values were expressed as mean ± SD. Results were analyzed by analysis of variance followed by a two-sided Dunnett's test or Student-Newman-Keuls test when appropriate. Linear correlation analysis was used for the relationship between TNF-*α* and VFs. Statistical significance was assumed at *P* < .05.

## 3. Results

### 3.1. TNF-*α* Expression Detected by Immunohistochemistry

**Figure 1:**
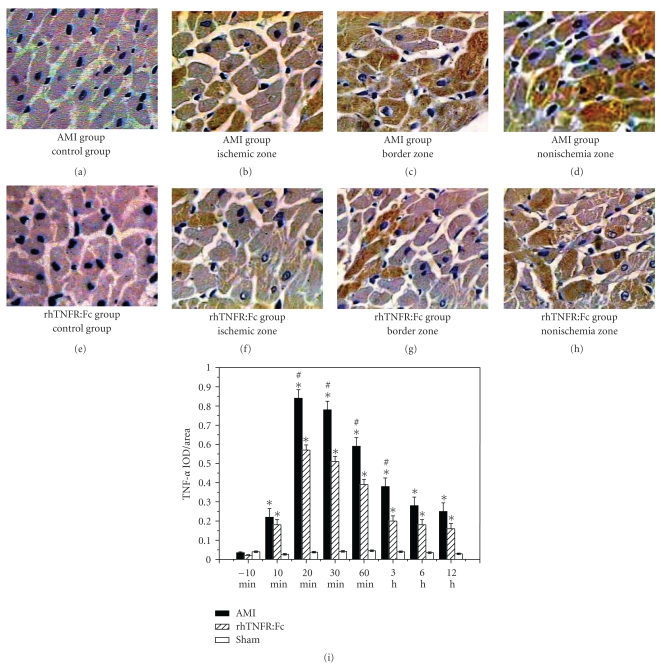
Expression of TNF-*α* detected by immunohistochemistry. (a)–(d) (×400) Expression of TNF-*α* in the control group, the ischemic zone, the border zone, and the non-ischemia zone of AMI group; (e)–(h) expression of TNF-*α* in the control group, the ischemic zone, the border zone, and the non-ischemia zone of rhTNFR:Fc group; (i) AMI group or rhTNFR:Fc group versus sham-operation group, *P < .05. AMI group versus rhTNFR:Fc group, ^#^ *P* < .05.

TNF-*α* in acute ischemic myocardium began to increase at 10 min after infarction, reached a climax at 20–30 min, and recovered gradually then. At the same time point, TNF-*α* in the ischemic zone was higher than the others, and the second was in the border zone. Compared to the AMI group, TNF-*α* detected by immunohistochemistry in rhTNFR:Fc group was significantly less than the AMI group (*P* < .05), and the expression of TNF-*α* in the sham group was extremely low (Figure 1(i)).

### 3.2. Expression of TNF-*α* mRNA Detected by Real-Time Fluorescence Quantitative PCR

**Figure 2:**
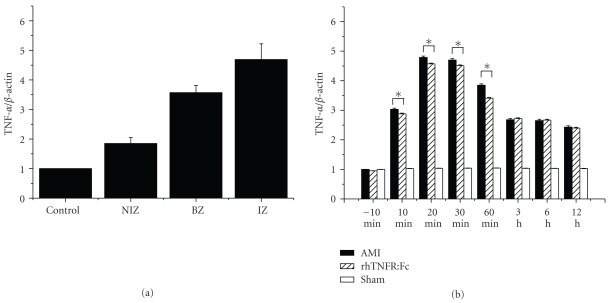
Expression of TNF-*α* mRNA detected by real-time fluorescent quantitative PCR. (a) Expression of TNF-*α* mRNA in the non-ischemia zone, the border zone, and the ischemic zone of AMI group; (b) expression of TNF-*α* mRNA among the different groups. AMI or rhTNFR:Fc group versus sham-operation group, *P < .05.

Expression of TNF-*α* mRNA in acute ischemic myocardium began to increase at 10 min after ligation, reached a peak time at 20–30 min, and recovered gradually then. At the same time point, the expression of TNF-*α* mRNA in the ischemic zone was higher than the others, and the second was the border zone. The expression of TNF-*α* mRNA in AMI group or rhTNFR:Fc group was higher than that in the sham group, *P* < .01; there was no significant difference between AMI group and rhTNFR:Fc group (Figure 2).

### 3.3. The Occurrence of VFs

**Figure 3:**
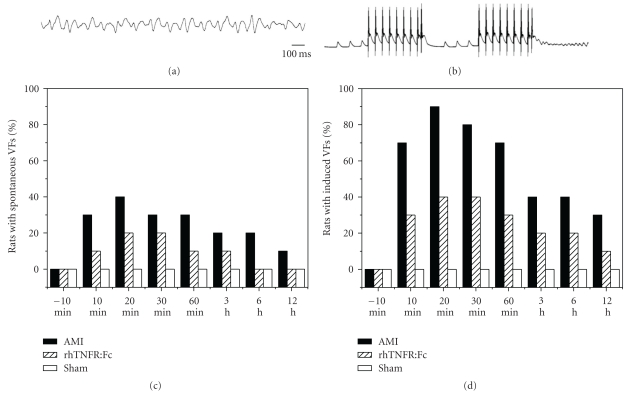
VFs caused by LAD ligation. (a) VF recorded by ECG; (b) VF recorded by MAP electrode. (c) Percentages of rats with spontaneous VFs; (d) percentages of rats with induced VFs.

In the AMI group, the spontaneous VFs appeared most frequently 10–30 min after ligation, reached a climax at 15–25 min, and recovered gradually then. Spontaneous VFs, which appeared on about 23% of AMI rats, generally lasted 0.8–1.5 s and automatically recovered. Three of them exceeded 3 min. Notwithstanding electrical defibrillation and manual compression, these VFs could not recover yet, and the rats died. In the AMI group, VFs can be induced by programmed electrical stimulation in 53% of rats, and the time window of induced VFs was similar to the spontaneous one. Compared to the AMI group, the occurrence of VFs in rhTNFR:Fc group significantly decreased, *P* < .05. There was no ventricular fibrillation in the sham group and the other two groups before ligation both in the induced and raw state. The occurrence of VFs was showed in Figure 3.

### 3.4. The Relationship between Expression of TNF-*α* and Induced VFs

**Figure 4:**
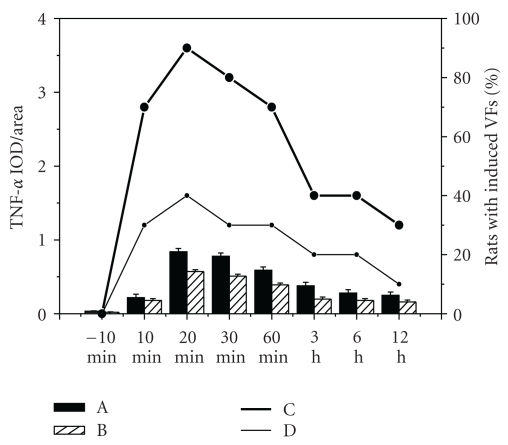
The relationship between TNF-*α* expression and induced VFs. A: Expression of TNF-*α* in AMI group; B: expression of TNF-*α* in rhTNFR:Fc group; C: percentages of rats with induced VFs in AMI group; D: percentages of rats with induced VFs in rhTNFR:Fc group.

**Figure 5:**
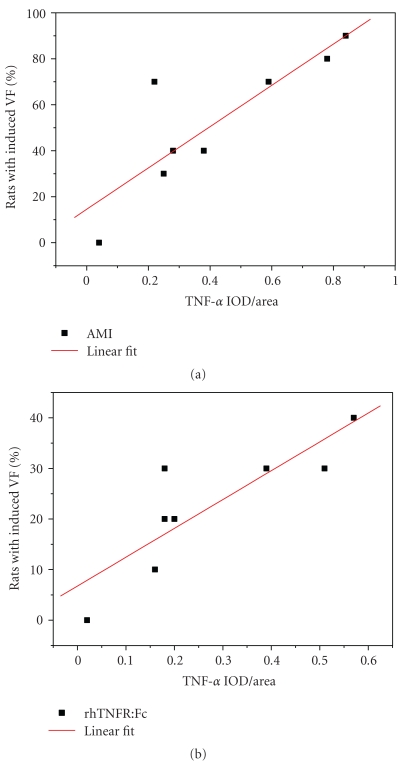
The correlation between TNF-*α* expression and VFs.

The time window of induced VFs was coincided with TNF-*α* expression in acute ischemic myocardium (Figure 4). Linear correlation analysis of the relationships between them was performed in both AMI group and rhTNFR:Fc group. The correlation coefficient of AMI group was 0.852, *P* < .01, the other one was 0.833, *P* < .01. Both showed positive linear correlation (Figure 5).

### 3.5. The Variation of the MAPDds in the Border Zone

**Figure 6:**
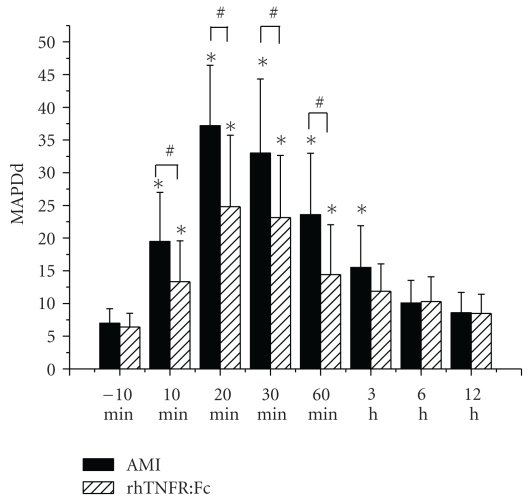
The variation of the MAPDds in the border zones. Compared to that before ligation, MAPDds in the border zone of the two groups after ligation significantly increased, *P < .01; MAPDds in the border zone of rhTNFR:Fc group were significantly less than AMI group, ^#^ *P* < .05.

The MAPDds in the border zone significantly increased after LAD ligation, reached a peak time at 20 min, and recovered gradually then (Figure 6). There was no obvious change both in the ischemic zone and the nonischemic zone. At the same time point, MAPDds of AMI group were significantly greater than rhTNFR:Fc group, *P* < .05. There was no obvious change in sham group. MAPDds of each group at different zones were shown in Table 1.

### 3.6. The Relationship between TNF-*α* Expression and MAPDds of the Border Zone

**Figure 7:**
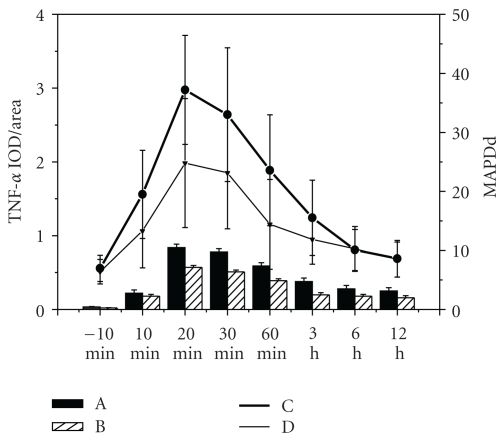
The relationship between TNF-*α* expression and MAPDds of the border zone. A: Expression of TNF-*α* in AMI group; B: expression of TNF-*α* in rhTNFR:Fc group; C: MAPDds of the border zone in AMI group; D: MAPDds of the border zone in rhTNFR:Fc group.

**Figure 8:**
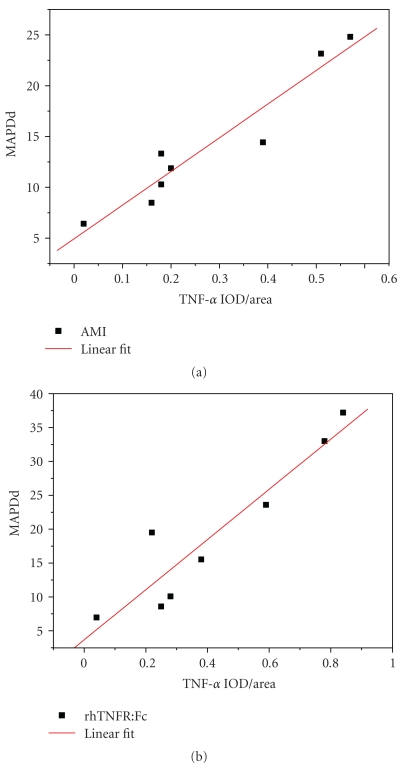
The correlation between TNF-*α* expression and MAPDds in the border zone.

The time window of the changes of MAPDds in the border zone was concerned to TNF-*α* expression in acute ischemic myocardium (Figure 7). Linear correlation analysis of the relationships between detected expression of TNF-*α* and the MAPDds in the border zone was performed both in AMI group and rhTNFR:Fc group. The correlation coefficient of AMI group was 0.935, *P* < .01, the other one was 0.959, *P* < .01. Both showed positive linear correlation (Figure 8).

## 4. Discussion

The study showed that expression of TNF-*α* in AMI group increased markedly by 10 min after infarction, reached a climax at 20–30 min, and recovered gradually then. The time window of VFs occurrence was similar to that of TNF-*α* expression, which showed that expression of TNF-*α* associated with VFs occurrence in AMI rats. With rhTNFR:Fc ahead of experiment, TNF-*α* detected in rhTNFR:Fc group was less than that in AMI group, and the incidence of VFs significantly reduced, suggesting that TNF-*α* promote the occurrence of VFs while rhTNFR:Fc effectively reduce the occurrence of VFs in AMI rats by antagonism to TNF-*α*.

The MAPDds in the border zone significantly increased after LAD ligation, reached climax at 20 min, and recovered gradually then. The time window of the TNF-*α* expression was similar to that of MAPDds in the border zone, which showed that MAPDds in the border zone were related to the expression of TNF-*α*. With rhTNFR:Fc, MAPDds in the border zone of rhTNFR:Fc group still increased, but the extent of increase sharply reduced. Meanwhile, the appearance of VFs significantly decreased as well, which suggested that TNF-*α* promotes the occurrence of acute ischemic VFs by enlarging MAPDds in the border zone, and rhTNFR:Fc decreases the occurrence of VFs by inhibiting the biological effects of TNF-*α*. 

Expression of TNF-*α* both in the border zone and the ischemic zone was high at the same time point, but only MAPDds in the border zone were significantly high. It was possibly due to the different sensitivity of the two areas to TNF-*α*. The ischemic extent of different myocardial cells in the border zone varied, and their physiological characteristics were different, so their response to the external stimulations (including TNF-*α*) was not the same too. In the ischemic zone, the ischemic extent of different myocardial cells was similar, their response to the external stimulations was identical, and, thus, there were no significant MAPDds.

The study is only a preliminary research into the possible role of TNF-*α* expression in the occurrence of VFs. Our findings suggested that TNF-*α* promotes acute ischemic VFs through enlarging MAPDds of the border zone. The underlying mechanisms need to be further studied. Besides, whether there are some other pathways in the process that TNF-*α* causes VFs remains unknown.

## 5. Conclusion

The expression of TNF-*α* increased greatly after acute myocardial infarction. TNF-*α* could enlarge the MADds in the border zone and promote the onset of VFs while rhTNFR:Fc could diminish the MADds in the border zone and lessen the onset of VFs in AMI rats. Our results demonstrated that TNF-*α* expressed by ischemic myocardium may play an important role in the occurrence of VFs in AMI rats.

## Figures and Tables

**Table 1:** MAPDds of different zones (cycle length = 167 ms).

Group	MAPDd	MAPDd (after ligation/ms)						
	(before ligation)	10 min	20 min	30 min	60 min	3 h	6 h	12 h
AMI group	6.97 ± 2.23							
IZ		10.83 ± 3.54	10.39 ± 2.88	9.72 ± 2.36	7.02 ± 2.45	6.57 ± 2.36	5.92 ± 1.26	5.40 ± 1.25
BZ		19.5 ± 7.48*	37.2 ± 9.24*	33.0 ± 11.32*	23.58 ± 9.4*	15.53 ± 6.37*	10.08 ± 3.46	8.59 ± 3.11
NIZ		10.28 ± 2.14	11.5 ± 2.47	9.72 ± 2.74	7.61 ± 2.09	7.53 ± 2.45	8.13 ± 2.7	7.11 ± 2.08
rhTNFR:Fc group	6.41 ± 2.08							
IZ		7.75 ± 3.22	8.42 ± 3.17	8.16 ± 2.23	6.59 ± 2.56	6.46 ± 2.04	7.1 ± 2.18	5.73 ± 1.59
BZ		13.32 ± 6.26^∗#^	24.8 ± 10.93^∗#^	23.15 ± 9.48^∗#^	14.42 ± 7.61^∗#∗^	11.87 ± 4.20	10.28 ± 3.80	8.47 ± 2.96
NIZ		8.51 ± 3.47	9.05 ± 3.62	9.33 ± 3.17	8.67 ± 2.92	6.40 ± 2.23	7.84 ± 2.74	5.52 ± 2.16
Sham group	7.68 ± 2.12	6.62 ± 1.78	7.4 ± 2.09	8.58 ± 2.43	7.1 ± 1.95	7.74 ± 2.29	6.28 ± 1.48	5.44 ± 1.37

MAPDds of the border zone in AMI group or rhTNFR:Fc group versus those in sham group, *P < .01. MAPDds in AMI group were greater than those in rhTNFR:Fc group, ^#^ *P* < .05.
